# Effects of socioeconomic position and clinical risk factors on spontaneous and iatrogenic preterm birth

**DOI:** 10.1186/1471-2393-14-117

**Published:** 2014-03-27

**Authors:** KS Joseph, John Fahey, Ketan Shankardass, Victoria M Allen, Patricia O’Campo, Linda Dodds, Robert M Liston, Alexander C Allen

**Affiliations:** 1 Department of Obstetrics and Gynaecology and the School of Population and Public Health, University of British Columbia and the Children’s and Women’s Hospital of British Columbia, 4500 Oak Street, Vancouver, British Columbia V6H 3 N1, Canada; 2 Reproductive Care Program of Nova Scotia, Halifax, Nova Scotia, Canada; 3 Department of Psychology, Wilfred Laurier University, Waterloo, Ontario, Canada; 4 Department of Obstetrics & Gynaecology, Dalhousie University and the IWK Health Centre, Halifax, Nova Scotia, Canada; 5 Centre for Research on Inner City Health, St. Michael’s Hospital and the University of Toronto, Toronto, Ontario, Canada; 6 Perinatal Epidemiology Research Unit, Departments of Obstetrics & Gynaecology and Pediatrics, Dalhousie University and the IWK Health Centre, Halifax, Nova Scotia, Canada; 7 Department of Obstetrics and Gynaecology, University of British Columbia, and the Children’s and Women’s Hospital of British Columbia, Vancouver, British Columbia, Canada

**Keywords:** Spontaneous preterm birth, Iatrogenic preterm birth, Risk factors, Pregnancy complications, Socioeconomic status

## Abstract

**Background:**

The literature shows a variable and inconsistent relationship between socioeconomic position and preterm birth. We examined risk factors for spontaneous and iatrogenic preterm birth, with a focus on socioeconomic position and clinical risk factors, in order to explain the observed inconsistency.

**Methods:**

We carried out a retrospective population-based cohort study of all singleton deliveries in Nova Scotia from 1988 to 2003. Data were obtained from the Nova Scotia Atlee Perinatal Database and the federal income tax T1 Family Files. Separate logistic models were used to quantify the association between socioeconomic position, clinical risk factors and spontaneous preterm birth and iatrogenic preterm birth.

**Results:**

The study population included 132,714 singleton deliveries and the rate of preterm birth was 5.5%. Preterm birth rates were significantly higher among the women in the lowest (versus the highest) family income group for spontaneous (rate ratio 1.14, 95% confidence interval (CI) 1.03, 1.25) but not iatrogenic preterm birth (rate ratio 0.95, 95% CI 0.75, 1.19). Adjustment for maternal characteristics attenuated the family income-spontaneous preterm birth relationship but strengthened the relationship with iatrogenic preterm birth. Clinical risk factors such as hypertension were differentially associated with spontaneous (rate ratio 3.92, 95% CI 3.47, 4.44) and iatrogenic preterm (rate ratio 14.1, 95% CI 11.4, 17.4) but factors such as diabetes mellitus were not (rate ratio 4.38, 95% CI 3.21, 5.99 for spontaneous and 4.02, 95% CI 2.07, 7.80 for iatrogenic preterm birth).

**Conclusions:**

Socioeconomic position and clinical risk factors have different effects on spontaneous and iatrogenic preterm. Recent temporal increases in iatrogenic preterm birth appear to be responsible for the inconsistent relationship between socioeconomic position and preterm birth.

## Background

Factors such as multi-fetal pregnancy, congenital malformations, older maternal age and race/ethnicity are strongly associated with preterm birth [1-4]. However, the relationship between socioeconomic position and preterm birth is variable and inconsistent, especially when compared with the relationship between socioeconomic position and small-for-gestational age (SGA) [5]. Further, the inconsistency in the relationship between socioeconomic position and preterm birth is evident irrespective of whether socioeconomic position is measured using income, education, occupation or area-based measures [5].

Another interesting aspect of the relationship between socioeconomic position and preterm birth is the documented temporal change in the association. For instance, in 1989, preterm birth rates among less educated women in the United States [6] were 73% (95% confidence interval (CI) 63, 83%) higher than preterm birth rates among more educated women, whereas in 2006 the excess risk was reduced to 31% (95% CI 22, 40%). A similar phenomenon was observed in New Zealand, where the strong socioeconomic gap in preterm birth rates observed in the 1980s disappeared by the turn of the century [7]. Some attenuation of the socioeconomic gap in preterm birth has also been observed in Denmark and Finland [8].

We hypothesized that the heterogeneity of preterm birth may constitute one potential explanation for the inconsistent and variable relation between socioeconomic position and preterm birth. It is possible that spatial and temporal variations in the frequency of the preterm birth subtypes, namely, spontaneous preterm birth (following preterm labour) and iatrogenic preterm birth (following labour induction or cesarean delivery before labour onset), or temporal changes in clinical risk factors (e.g., older maternal age and complications of pregnancy) for these subtypes of preterm birth underlie the vagaries of the socioeconomic position–preterm birth relationship.

The Canadian setting is likely ideal for examining potential changes in the relationship between socioeconomic position, obstetric intervention and preterm birth subtypes, given universal health insurance coverage for obstetric and related medical services. We therefore carried out a study to examine the determinants of spontaneous and iatrogenic preterm birth, with a focus on the effect of socioeconomic position and clinical risk factors.

## Methods

The study cohort included all live births and stillbirths to residents of Nova Scotia, Canada between 1988 and 2003. Detailed information for the study was obtained from the Nova Scotia Atlee Perinatal Database, a population-based database which contains information on maternal characteristics, labor and delivery events, and neonatal diagnoses and procedures. The database is collated from information abstracted from antenatal and medical charts by trained personnel using standardized forms. An ongoing data-quality assurance program, which conducts periodic abstraction studies, and validation studies [9,10] have shown the data to be accurate and reliable.

Births with a birth weight <500 g or a gestational age <20 weeks were excluded to avoid potential bias due to changes in the registration of births at the borderline of viability [11,12]. A confidential linkage between the Nova Scotia Atlee Perinatal Database (1988 to 2003) and federal income tax (T1 Family File) records (1988 to 2003) was carried out to supplement the clinical information in the database with information on socioeconomic position (reported previously [13]). The T1 Family Files, which are maintained by the Small Area and Administrative Data Division of Statistics Canada in Ottawa, were created from several administrative data sources, with income tax returns serving as the primary source [14]. The linkage was carried out using deterministic and probabilistic methods and resulted in a successful linkage of 135,945 of 167,187 (81.3%) of the births. All linkages and analyses were carried out by Small Area and Administrative Data Division personnel in secure offices. Tabular analyses involving income and related information that resulted in cells with fewer than 15 were suppressed, and all tabulated counts were rounded to the nearest 10. However, regression analyses were carried out without such restrictions.

Annual after-tax family income for each woman (in the year of delivery) was based on the T1 Family Files and adjusted for family size. The latter calculation was carried out by dividing the total family income by the weighted number of family members. Weights were based on a standard formula (the oldest adult in the family received a weight of 1, other members 16 years or older and the first child in a single parent family received a weight of 0.4, and children under 16 years received a weight of 0.3) [15]. Family income was also adjusted for inflation (expressed in 1988 Canadian dollars) and categorized to yield five approximately equal groups. The quintile with the lowest family income was then further subdivided (in order to obtain a potentially more vulnerable group) and this resulted in a total of six family income categories (< $4,430, $4,430–$6,529, $6,530–$11,914, $11,915–$17,584, $17,585–$24,949, and ≥ $24,950). Contribution to a retirement savings plan (RSP, a tax-deductible investment) in the year of delivery was also examined as a second measure of socioeconomic position. This measure was used as an indicator of behavior related to tax-planning and long-term saving and was expected to reflect a dimension of socioeconomic status that was distinct from family income [16].

Rates of preterm birth <37 completed weeks gestation and preterm birth <32 completed weeks among singletons (N = 132,714) were examined within categories of family income and contribution to a retirement savings plan. Gestational age was based on the duration between the last menstrual period and the delivery if it was consistent with the neonatal physical examination of the infant and based on the neonatal physical examination in cases where the discrepancy between the 2 methods of gestational age ascertainment was 3 weeks or greater. Preterm births were categorised as iatrogenic if labor was induced or if a cesarean delivery was carried out in the absence of labor, and as spontaneous otherwise.

The crude and adjusted effects of socioeconomic position and other factors on spontaneous/iatrogenic preterm birth were examined in the study population using 3 different logistic models. All models accounted for the non-independence of observations among births to the same women using generalized estimating equations. The first of the three models provided the crude effects of socioeconomic position and included family income and contribution to a retirement savings plan as the independent variates and spontaneous (or iatrogenic) preterm birth as the dependent variate. The second set of logistic models were used to assess the effect of family income and contribution to a retirement savings plan after adjustment for extraneous maternal characteristics that could have confounded the socioeconomic position-spontaneous/iatrogenic preterm birth relation (namely, maternal age, parity, marital status, residence, pre-pregnancy weight, smoking status, previous cesarean delivery, previous low birth weight, previous perinatal death and calendar period). The last of the 3 models also included medical complications of pregnancy (gestational hypertension, pre-existing hypertension, gestational diabetes, diabetes mellitus, other chronic medical disease, placenta previa and placental abruption). Other chronic medical disease included cardiovascular disorders, renal disease, gastrointestinal disorders, respiratory disease, endocrine disorders, neurologic disease, blood dyscrasias and miscellaneous illnesses. This most proximal set of potential confounders was added last because some potentially lie in the causal pathway between maternal characteristics and spontaneous/iatrogenic preterm birth. Since rates of preterm birth and its subtypes (e.g., spontaneous/iatrogenic preterm, preterm birth <32 weeks) were rare, the odds ratios from the logistic models were considered to approximate proportion type rate ratios. The statistical significance of differences in the odds ratios expressing the effect of a risk factor on spontaneous preterm birth vs iatrogenic preterm birth was assessed using a Chi-square test for the heterogeneity [17]. Analysis was carried out using SAS software (Cary, NC) and the study was approved by the IWK Health Centre Research Ethics Board.

## Results

**Figure 1:**
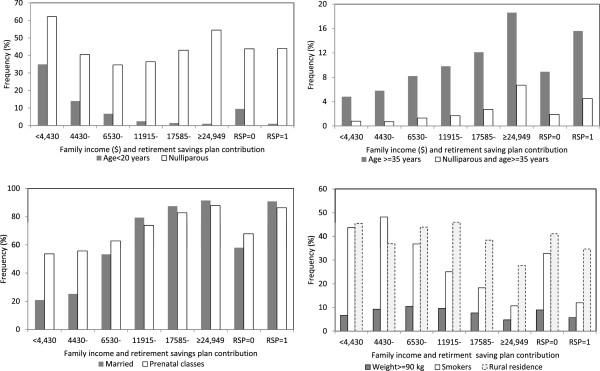
Maternal characteristics by family income and contribution to a retirement savings plan (RSP) among singleton births, Nova Scotia, 1988–2003 (rates of prenatal class attendance restricted to nulliparous women).

Maternal characteristics such as age, parity, marital status, rural residence, pre-pregnancy weight and smoking status varied by family income and RSP status (Figure 1). For instance, 34.9% of mothers in the lowest family income category were <20 years age compared with 1.0% of mothers in the highest family income group (Table 1). On the other hand, 18.6% of mothers in the highest family income group were *> =* 35 years of age compared with only 4.8% of mothers in the lowest family income category. Such differences were also evident in contrasts by RSP contribution in the year of delivery (Table 1).

**Table 1:** Maternal characteristics by family income and contribution to a retirement savings plan (RSP) among singleton births, Nova Scotia, 1988-2003

**Characteristic**	Family income*						**RSP (no)**	**RSP (yes)**
	**<$4,430**	**$4,430 to $6,529**	**$6,530 to $11,914**	**$11,915 to $17,584**	**$17,585 to $24,949**	**≥$29,450**		
	N = 13,217	N = 13,215	N = 26,354	N = 26,536	N = 26,628	N = 26,764	N = 96,356	N = 36,358
Maternal age <20 yrs (%)	34.9	14.0	6.7	2.4	1.4	1.0	9.5	1.0
Maternal age ≥35 yrs (%)	4.8	5.8	8.2	9.8	12.1	18.6	8.9	15.6
Nulliparous (%)	62.1	40.5	34.6	36.4	43.0	54.5	43.8	44.0
Nulliparous, ≥35 yrs (%)	0.8	0.7	1.3	1.7	2.7	6.7	1.9	4.5
Married (%)	20.9	25.2	53.3	79.3	87.4	91.5	58.0	90.8
Rural residence (%)	45.4	36.9	43.9	46.0	38.4	27.7	41.1	34.7
Prenatal classes^†^ (%)	53.6	55.7	62.8	73.8	82.7	87.8	67.8	86.3
Pre-preg. Wt. ≥90 kg (%)	6.7	9.3	10.5	9.6	7.7	4.8	9.0	5.7
Delivery Wt. ≥100 kg (%)	10.0	12.2	13.2	12.3	10.8	7.5	11.9	8.4
Smokers (%)	43.7	48.2	36.8	25.1	18.3	10.7	32.8	12.0

*Family income adjusted for family size and expressed in 1988 Canadian dollars.

^†^Prenatal class attendance among nulliparous women only.

The overall rate of preterm birth (<37 weeks) was 5.5%; the rate of spontaneous preterm birth was 4.7% and the rate of iatrogenic preterm birth was 0.8%. Crude rates of preterm birth <37 weeks were significantly higher among women with the lowest family income (6.1%) compared with women with the highest family income (5.6%, Table 2). Logistic regression adjustment for known determinants of preterm birth abolished this association. Rates of preterm birth <32 weeks gestation were not significantly different by family income; adjustment for known determinants did not change this association. The crude relationships between RSP contribution and preterm birth <37 weeks and preterm birth <32 weeks were statistically significant; logistic regression adjustment attenuated both these relationships.

**Table 2:** Rates of preterm birth <37 weeks and preterm birth <32 weeks and crude and adjusted rate ratios by family income and contribution to a retirement saving plan (RSP) among singletons births, Nova Scotia, 1988 to 2003

Family income*/RSP	**Preterm birth**		**Crude rate ratio**	**95% CI**	**P value**	**Adjusted rate ratio**^**‡**^	**95% CI**	**P value**
	**Number**^**†**^** Rate (%)**^**†**^							
Preterm birth <37 weeks								
<$4,430	780	6.1	1.11	1.01-1.21	0.03	1.01	0.88-1.16	0.87
$4,430-$6,529	800	6.2	1.12	1.02-1.22	0.02	1.03	0.90-1.18	0.69
$6,530-$11,914	1,390	5.4	0.97	0.90-1.05	0.47	0.96	0.86-1.07	0.44
$11,915-$17,584	1,270	5.1	0.90	0.83-0.97	0.007	0.97	0.87-1.07	0.52
$17,585-$24,949	1,390	5.5	0.98	0.91-1.06	0.61	1.07	0.97-1.17	0.17
$ ≥ 29,450	1,380	5.6	1.00	-	-	1.00	-	-
RSP (no)	5,240	5.7	1.11	1.05-1.18	0.0001	1.08	1.00-1.16	0.06
RSP (yes)	1,780	5.1	1.00	-	-	1.00	-	-
Preterm birth <32 weeks								
<$4,430	100	0.86	1.14	0.90-1.43	0.29	0.74	0.50-1.10	0.14
$4,430-$6,529	110	0.92	1.21	0.96-1.52	0.11	0.87	0.59-1.28	0.47
$6,530-$11,914	190	0.79	1.04	0.85-1.26	0.72	0.82	0.60-1.11	0.19
$11,915-$17,584	170	0.75	0.98	0.80-1.20	0.86	0.90	0.68-1.18	0.44
$17,585-$24,949	170	0.72	0.95	0.78-1.16	0.62	0.92	0.72-1.19	0.52
$ ≥ 29,450	150	0.76	1.00	-	-	1.00	-	-
RSP (no)	670	0.81	1.18	1.02-1.37	0.02	1.17	0.94-1.44	0.15
RSP (yes)	210	0.69	1.00	-	-	1.00	-	-

*Family income adjusted for family size and expressed in 1988 Canadian dollars.

^†^All numbers rounded to the nearest 10 in tabular analyses, with crude rates (and crude rate ratios) obtained from logistic models (excluding those with a missing gestational age).

^‡^Adjusted rate ratios were obtained from a logistic model that included maternal age, parity, marital status, residence, pre-pregnancy weight, smoking status, previous cesarean delivery, previous low birth weight, previous perinatal death and calendar period.

The crude relationship between family income and spontaneous preterm birth was complex (Table 3). The crude rate ratio for spontaneous preterm birth was significantly higher among the women in the lowest 2 family income groups (<$4,430 and $4,430 to $6,529) compared with women in the highest family income group (*> =* $24,950). Also, women in the middle family income category ($11,915-$17,584) had a significantly lower rate of spontaneous preterm birth compared with women in highest family income group. Finally, women who did not contribute to an RSP also had a 14% (95% CI 8, 21) higher rate of spontaneous preterm birth compared with women who contributed to an RSP. On the other hand, the crude relationships between family income and iatrogenic preterm birth, and the crude relationship between contribution to an RSP and iatrogenic preterm birth were not statistically significant (Table 3).

**Table 3:** Crude association between family income and contribution to a retirement savings plan (RSP) and subtypes of preterm birth (<37 weeks) among singleton births, Nova Scotia, Canada, 1988 to 2003

Family income*/RSP	**Spontaneous preterm birth**			**Iatrogenic preterm birth**		
	**Crude rate ratio**	**95% CI**	**P value**	**Crude rate ratio**	**95% CI**	**P value**
<$4,430	1.14	1.03-1.25	0.009	0.95	0.75-1.19	0.65
$4,430-$6,529	1.14	1.04-1.26	0.006	0.98	0.78-1.23	0.88
$6,530-$11,914	0.99	0.91-1.07	0.75	0.92	0.76-1.11	0.36
$11,915-$17,584	0.91	0.84-0.99	0.02	0.86	0.71-1.04	0.11
$17,585-$24,949	1.00	0.92-1.08	0.97	0.88	0.73-1.07	0.20
$ ≥ 29,450	1.00	-	-	1.00	-	-
No RSP	1.14	1.08-1.21	<0.0001	0.98	0.86-1.12	0.76
RSP	1.00	-	-	1.00	-	-

*Family income adjusted for family size and expressed in 1988 Canadian dollars.

Table 4 contrasts the results of logistic regression for spontaneous preterm birth and iatrogenic preterm birth. Adjustment for other determinants of preterm birth abolished the crude relationship between low family income and spontaneous preterm birth (crude rate ratio for family income < $4,430 = 1.14, 95% CI 1.03, 1.25, Table 3; adjusted rate ratio = 0.99, 95% CI 0.85, 1.15, Table 4). On the other hand, adjustment increased the rate ratio between low family income and iatrogenic preterm birth, although neither the crude nor the adjusted effect was statistically significant (crude rate ratio for family income < $4,430 = 0.95, 95% CI 0.75, 1.19; adjusted rate ratio 1.12, 95% CI 0.77, 1.62). No RSP contribution was a risk factor for spontaneous preterm birth in the crude model and adjustment for maternal characteristics attenuated this relationship. These crude and adjusted associations between RSP contribution and iatrogenic preterm birth were not significant (Table 4).

**Table 4:** Factors associated with spontaneous and iatrogenic preterm birth (<37 weeks) among singleton births, Nova Scotia, Canada, 1988 to 2003

**Determinant**	**Spontaneous preterm birth**			**Iatrogenic preterm birth**		
	**Adjusted rate ratio**	**95% CI**	**P value**	**Adjusted rate ratio**	**95% CI**	**P value**
Family income* < $4,430	0.99	0.85-1.15	0.92	1.12	0.77-1.62	0.56
$4,430-$6,529	1.01	0.87-1.17	0.89	1.13	0.79-1.61	0.51
$6,530-$11,914	0.95	0.84-1.07	0.42	0.99	0.74-1.32	0.94
$11,915-$17,584	0.95	0.86-1.06	0.40	1.03	0.80-1.32	0.81
$17,585-$24,949	1.07	0.97-1.19	0.16	1.00	0.79-1.26	0.97
≥$24,950	1.00	-	-	1.00	-	-
RSP (No)	1.09	1.00-1.18	0.05	1.02	0.84-1.23	0.84
Age <20 years	0.96	0.84-1.10	0.59	0.85	0.57-1.27	0.42
20-24	1.00	-	-	1.00	-	-
25-29	1.13	1.03-1.24	0.009	1.37	1.07-1.76	0.01
30-34	1.34	1.21-1.49	<0.0001	1.46	1.11-1.92	0.007
35-39	1.54	1.35-1.77	<0.0001	2.02	1.46-2.80	<0.0001
≥40	1.51	1.13-2.03	0.006	4.47	2.77-7.22	<0.0001
Parity 0	1.98	1.83-2.14	<0.0001	2.63	2.12-3.26	<0.0001
1	1.00	-	-	1.00	-	-
2	0.84	0.74-0.94	0.003	1.02	0.80-1.31	0.88
≥3	0.81	0.68-0.96	0.02	1.04	0.73-1.49	0.82
Marital status: Single	1.12	1.01-1.24	0.03	0.92	0.71-1.20	0.56
Married	1.00	-	-	1.00	-	-
Common law	1.17	1.05-1.30	0.006	0.95	0.72-1.26	0.74
Other	1.25	0.96-1.63	0.09	1.30	0.75-2.23	0.35
Rural residence (Yes)	1.01	0.94-1.07	0.88	0.87	0.74-1.02	0.09
Weight <55 kg	1.33	1.22-1.45	<0.0001	0.97	0.78-1.21	0.80
55-59	1.12	1.02-1.23	0.02	0.92	0.73-1.16	0.48
60-69	1.00	-	-	1.00	-	-
70-79	1.05	0.95-1.16	0.35	1.04	0.82-1.31	0.75
80-89	1.07	0.94-1.22	0.33	0.91	0.67-1.22	0.52
≥90	1.15	1.01-1.31	0.04	1.21	0.92-1.60	0.17
Smoking: No	1.00	-	-	1.00	-	-
Yes 1–9 cig.	1.29	1.15-1.44	<0.0001	1.22	0.92-1.63	0.17
Yes ≥10 cig.	1.28	1.18-1.39	<0.0001	1.23	1.00-1.52	0.05
Prev. cesarean	0.83	0.73-0.94	0.003	4.91	3.96-6.08	<0.0001
Prev. low birth wt.	4.11	3.56-4.75	<0.0001	2.94	2.18-3.97	<0.0001
Prev. perinatal death	1.31	1.01-1.70	0.04	1.96	1.26-3.05	0.003
Period: 1988-1990	1.00	-	-	1.00	-	-
1991-1993	1.00	0.91-1.10	0.98	1.09	0.85-1.40	0.49
1994-1996	1.00	0.91-1.10	0.99	1.34	1.06-1.71	0.02
1997-1999	1.15	1.04-1.26	0.005	1.35	1.06-1.72	0.01
2000-2003	1.09	0.98-1.20	0.10	1.60	1.26-2.02	0.0001

*Family income adjusted for family size and expressed in 1988 Canadian dollars. RSP denotes a Registered Saving Plan (see text).

Spontaneous and iatrogenic preterm birth had several risk factors in common and some that were distinct. Older maternal age was a risk factor for both spontaneous and iatrogenic preterm birth, although the magnitude of the association was significantly stronger for iatrogenic preterm birth. For instance, women *> =* 40 years had a 1.51 (95% CI 1.13, 2.03) times greater risk of spontaneous preterm birth compared with women 20–24 years, while women *> =* 40 years had a 4.47 (95% CI 2.77, 7.22) times greater risk of iatrogenic preterm birth. These two rate ratios were significantly different from each other (P < 0.05 for heterogeneity). Nulliparous women were at higher risk of both spontaneous and iatrogenic preterm birth compared with women of parity 1, though again the excess risk was higher with iatrogenic preterm birth. However, parity *> =* 3 was protective against spontaneous preterm birth but not significantly related to iatrogenic preterm birth (Table 4).

Other differences in risk factors for spontaneous and iatrogenic preterm birth included marital status. Single women and women in a common law relationship had higher rates of spontaneous preterm birth compared with married women, while marital status did not influence rates of iatrogenic preterm birth (Table 4). Women with a low or high pre-pregnancy weight (<55 kg, 55–59 kg and *> =* 90 kg) had an increased risk of spontaneous preterm birth compared with women with a pre-pregnancy weight of 60–69 kg. However, pre-pregnancy weight was not associated with iatrogenic preterm birth. Maternal smoking was associated with both spontaneous and iatrogenic preterm birth to a similar degree, while a previous cesarean delivery was a protective factor for spontaneous preterm birth (rate ratio 0.83, 95% CI 0.73, 0.94) and a strong risk factor for iatrogenic preterm birth (rate ratio 4.91, 95% CI 3.96, 6.08; P value for heterogeneity <0.05). Previous low birth weight and previous perinatal death were similarly associated with spontaneous and iatrogenic preterm birth. Spontaneous preterm birth rates showed an increasing trend over calendar time but the rate in 2000–2003 was not significantly higher than the rate in 1988–1990 (adjusted rate ratio 1.09, 95% CI 0.98, 1.20). On the other hand, rates of iatrogenic preterm birth increased steadily and the adjusted rate in 2000–2003 was 60% (95% CI 26, 102) higher than the rate in 1988–1990.

Associations between maternal complications and the two subtypes of preterm birth showed distinct differences (Table 5). Gestational diabetes and other chronic medical disease were weakly or moderately associated with spontaneous preterm birth, while hypertension, diabetes mellitus, placenta previa and abruption were strongly associated. On the other hand, gestational diabetes was not associated with iatrogenic preterm birth, while the association between hypertension and iatrogenic preterm birth and between placenta previa and iatrogenic preterm birth was much stronger than the corresponding associations with spontaneous preterm birth (P value for heterogeneity of the odds ratio <0.05). The magnitude of the association between diabetes mellitus, other chronic medical disease and placental abruption, and the two subtypes of preterm birth was similar.

**Table 5:** Association between maternal complications of pregnancy and subtypes of preterm birth among singleton births, Nova Scotia, Canada,1988 to 2003

**Maternal complication**	**Spontaneous preterm birth**			**Iatrogenic preterm birth**		
	Adjusted rate ratio^*^	**95% CI**	**P value**	Adjusted rate ratio^*^	**95% CI**	**P value**
Hypertension	3.92	3.47-4.440	<0.0001	14.1	11.4-17.4	<0.0001
Gestational diabetes	1.83	1.56-2.15	<0.0001	0.92	0.59-1.43	0.70
Diabetes	4.38	3.21-5.99	<0.0001	4.02	2.07-7.80	<0.0001
Chronic med. disease	1.34	1.16-1.55	<0.0001	1.58	1.16-2.15	0.003
Placenta previa	2.92	2.00-4.27	<0.0001	82.8	59.6-115.2	<0.0001
Placental abruption	9.15	7.78-10.8	<0.0001	12.6	9.14-17.5	<0.0001

*Adjusted rate ratios were obtained from a logistic model that included family income, contribution to a registered savings plan, maternal age, parity, marital status, residence, pre-pregnancy weight, smoking status, previous cesarean delivery, previous low birthweight, previous perinatal death and calendar period. Chronic medical disease included cardiovascular disorders, renal disease, gastrointestinal disorders, respiratory disease, endocrine disorders, neurologic disease, blood dyscrasias and miscellaneous illnesses.

Supplementary analyses showed that women whose perinatal records linked to socioeconomic information in the T1 Family Files (81.3%) were generally similar to women whose records did not link (18.7%); 10.4% of women whose records linked were *> =* 35 years compared with 8.7% whose records did not link. Similarly, of the women whose records linked to socioeconomic information 43.8% were nulliparous, 7.5% had a pre-pregnancy weight > = 90 kg, 63.3% were married and 39.4% lived in a rural area compared with 46.6%, 6.8%, 63.3% and 39.2%, respectively, of women whose records did not link. The rate of preterm birth among women with unlinked records was 6.0% (compared with 5.5% among women with linked records).

## Discussion

Our study shows that spontaneous and iatrogenic preterm birth have several common and distinct risk factors. Family income and contribution to a retirement savings plan were associated with crude rates of spontaneous preterm birth but not with crude rates of iatrogenic preterm birth. Similarly, factors such as older maternal age, parity and previous cesarean delivery had significantly different effects on spontaneous and iatrogenic preterm birth. Adjustment for determinants of preterm birth attenuated the socioeconomic associations with spontaneous preterm birth and strengthened associations with iatrogenic preterm birth. Finally, adjusted temporal trends in spontaneous and iatrogenic preterm birth were distinct, with a small, non-significant increase in spontaneous preterm birth and a substantial, significant increase in iatrogenic preterm birth.

These data provide some insight into the observed temporal attenuation in the relationship between socioeconomic position and preterm birth seen in several countries [6,7]. Since older maternal age is associated with spontaneous preterm birth and more strongly associated with iatrogenic preterm birth, the differential temporal increases in older maternal age by socioeconomic position [13] would have led to increases in rates of preterm birth among more affluent women. This in turn would have resulted in a diminution in the gradient between socioeconomic position and preterm birth. Other factors that appear to have been responsible for the temporal change in the relation between socioeconomic status and preterm birth include the increase in the proportion of women with a previous cesarean delivery given the strong association between this factor and iatrogenic preterm birth. This underscores the complex nature of the various behavioral and lifestyle mediators responsible for socioeconomic gradients in preterm birth [18] and in health more generally.

The overlap between the etiology of spontaneous and iatrogenic preterm birth has been previously described [19-24]. Indications for obstetric intervention at preterm gestation (including preeclampsia, placental abruption, growth restriction and fetal compromise) are also risk factors for spontaneous preterm birth. In fact, with recent improvements in fetal surveillance and monitoring, iatrogenic preterm birth and spontaneous preterm birth are clearly competing risks in any high risk pregnancy. It is likely that the anticipated temporal increase in spontaneous preterm birth (secondary to temporal increases in older maternal age and multi-fetal pregnancy) was moderated by the observed substantial increase in iatrogenic preterm birth.

Our study shows that crude rates of spontaneous preterm birth are associated with indices of socioeconomic position including family income and contribution to a retirement savings plan. Adjustment for maternal characteristics such as age, parity, marital status, pre-pregnancy weight and smoking abolished or substantially attenuated these associations strongly suggesting that such lifestyle factors mediate the excess risk of spontaneous preterm birth by socioeconomic position. On the other hand, family income and contribution to a retirement savings plan were not associated with iatrogenic preterm birth. However, this is not unexpected in the Canadian setting where universal coverage for obstetric and related medical services is available under the Canadian health care system [25,26].

The overall rate of preterm birth in our study was low (5.5%) and the proportion due to iatrogenic preterm birth was also low (rate 0.8% i.e., 15% of preterm births). This is because the study was restricted to singleton births for the period 1988 to 2003. For instance in 1997, the overall rate of preterm birth in Canada was 7.1% and among singletons, the preterm birth rate was 5.9% [27]. A previous study from Nova Scotia also reported a preterm birth rate of 5.5% and an iatrogenic preterm birth rate of 1.4% for the period 1995–97 among live births without congenital malformations [28]. Differences between this study [28] (which excluded infants with congenital malformations and included multiple births) and ours, make these rates approximately similar (the rate of preterm induction and preterm cesarean delivery among twins in Nova Scotia in 1996–97 was 8.6% and 23.9%, respectively) [29].

The strengths of our study include the use of information from two high quality databases. The Nova Scotia Atlee Perinatal Database had detailed information that permitted the classification of preterm birth subtypes based on labor characteristics (presence/absence of labor and whether labor was induced or spontaneous). Similarly, information from the federal T1 Family Files and calculation of family income (adjusted for family size and inflation) permitted an accurate estimate of socioeconomic position. The use of contribution to a retirement savings plan as an index of socioeconomic behavior that was distinct from family income was another strength of the study.

The limitations of our study included those that are typically associated with information in large databases. Some accuracy may have been compromised due to transcription and related errors. Also, height was not available for calculation of body mass index, and gestational age was assessed based on menstrual dating confirmed by neonatal physical examination. Although we did not have ultrasound confirmed gestational age, the same uniform algorithm for gestational age assessment was used over the entire duration of the study from 1988 to 2003. Finally, our population-based data source, which included all singleton births in Nova Scotia (≥20 weeks gestation or ≥500 g), was affected by our inability to link all perinatal records with T1 Family File socioeconomic information. However, the linkage rate was high (81.3%) and there were only modest differences in maternal characteristics between women whose records did and did not link. Finally, we only examined one dimension of the heterogeneity of preterm birth (spontaneous vs iatrogenic), although several other classification schemes have been previously described in the literature [30-32]. This was because our hypothesis was based on the potential alteration in the relationship between socioeconomic position and preterm birth (because of increases in iatrogenic preterm birth).

## Conclusions

Our study showed that socioeconomic position was associated with crude rates of spontaneous preterm birth but not with crude rates of iatrogenic preterm birth. Older maternal age, parity, previous cesarean delivery and pregnancy complications had significantly different effects on spontaneous and iatrogenic preterm birth. Adjusted temporal trends in spontaneous and iatrogenic preterm birth were distinct, with a small non-significant increase in spontaneous preterm birth and a substantial and significant increase in iatrogenic preterm birth. These findings explain inconsistencies in the literature with regard to the relationship between socioeconomic position and preterm birth and the observed changes in this relationship.

## Competing interest

The authors declare they have no competing interests.

## Authors’ contributions

The study was based on a grant proposal submitted by the authors and funded by the Canadian Institutes of Health Research. Analyses were carried out by KSJ who submitted SAS programs to the office of the Small Area and Administrative Data Division of Statistics Canada in Ottawa. All authors reviewed the preliminary and final analyses, and the draft and final manuscripts. All authors read and approved the final manuscript.

## Pre-publication history

The pre-publication history for this paper can be accessed here:

http://www.biomedcentral.com/1471-2393/14/117/prepub
